# Stability and Success of Clear Aligners in Orthodontics: A Narrative Review

**DOI:** 10.7759/cureus.52038

**Published:** 2024-01-10

**Authors:** Hattan S Katib, Areej M Hakami, Mashail Albalawei, Saif A Alhajri, Mishal S Alruwaily, Moath I Almusallam, Ghaida H Alqahtani

**Affiliations:** 1 Orthodontics, King Fahd Hospital, Madinah, SAU; 2 General Dentistry, Al-Kadi Medical Centre, Tabuk, SAU; 3 General Dentistry, Tabuk National Dental Center, Tabuk, SAU; 4 General Dentistry, Al Safwa Saudi Medical Company, Riyadh, SAU; 5 General Dentistry, Imam Abdulrahman Bin Faisal University, Dammam, SAU; 6 General Dentistry, Al-Farabi College for Nursing and Dentistry, Jeddah, SAU

**Keywords:** invisalign, orthodontic patients, orthodontic management, clear aligner, orthodontic

## Abstract

The implementation of Clear Aligner Therapy (CAT) in adult orthodontics exemplifies the integration of advanced technology in the dental healthcare sector. Representing a significant shift in modern orthodontics, CAT offers a convenient and aesthetic alternative to traditional fixed appliance treatments for mal-aligned dentition. This narrative review aims to explore the applicability of CAT, delineating its biomechanics, indications, contraindications, scope, limitations, and factors influencing long-term stability and successful outcomes. A comprehensive literature search was conducted using databases like Google Scholar, PubMed, Cereus, and the Cochrane Library. Articles were selected based on their relevance to clear aligners, without brand specificity, and covered a wide range of cases to establish CAT’s scope and limitations. This review includes individual case studies, systemic reviews, comparative analyses, case reports, finite element analyses, and prospective and retrospective analyses, all contributing to a nuanced understanding of CAT’s applicability and long-term treatment stability. The conclusion underscores CAT’s growing acceptance in orthodontics, including its application in challenging cases, and highlights key determinants that bolster its long-term efficacy.

## Introduction and background

The world is continuously progressing, and the dental healthcare setup is also experiencing the introduction of novelties and advanced technology for a long time. The implementation of Clear Aligner Treatment (CAT) in adult orthodontics is one such example. Clear aligners represent a paradigm shift in modern orthodontics, providing a more convenient solution for repositioning mal-aligned dentition than conventional fixed orthodontic treatment. These thin plastic devices, made from semi-elastic polyurethane [1], are aesthetic, comfortable, and facilitate good oral hygiene maintenance, making them a preferable substitute to conventional metallic brackets [2], owing to the higher patient satisfaction rate and refinement of occlusal functionality [3].

They have undergone a series of evolutions to look and function as they do today. The history dates back to the origin of the tooth positioner system in orthodontics by Keslings in 1945, as an optimization appliance used in the final stages of treatment after de-banding [4]. The operational efficacy extended, and the ideation was carried forward and underwent sequential developments until 1998 when the advancements in CAT by Align Technology Inc. (San Jose, California, United States) took over the market [5].

The ongoing metamorphosis is what we are experiencing in present times; the integration of transparent thermoplastic materials, digitalization with Computer-Aided Design/Computer-Aided Manufacturing (CAD/CAM), stereolithography, and tooth-movement simulation software, i.e., ClinCheck® Pro software (Align Technology, Inc.). These breakthroughs transformed the course of treatment. Initially, their use was confined to minor tooth discrepancies and mild malocclusions; clear aligners were considered beneficial for partial tooth movements, leading to rapid case completion, whereas braces remained advantageous against a wide variety of tooth movements including root paralleling and torquing, and less relapse [6]. However, the incorporation of various auxiliaries and attachments with different biomechanics and unique construction methods has made it possible to treat complex cases with successful outcomes [2], which is further studied in this review. Moreover, some of the other positive features of CAT included good periodontal health due to accessible teeth and gum care, as the appliances are removable [7], and fewer appointments and emergency visits compared to braces [8].

The applicability of clear aligners has been studied in a variety of cases, but further investigation is needed to fully comment on their potential and wider scope. The long-term stability followed by a complete course of treatment with CAT accounts for its success, predictability, and effectiveness. Above all, the chances of relapse determine the prognosis of the outcome.

This review aims to critically assess the applicability, limitations, and scope of CAT. It endeavors to link the outcomes from various malocclusion treatments to evaluate the overall stability of treatment with clear aligners. Furthermore, the review will identify key determinants that contribute to the long-term success of this treatment modality.

## Review

Clear aligner biomechanics

As a removable appliance, clear aligners are most efficient at executing the simplest of all tooth movements, such as tipping. Their effectiveness in root uprighting in extraction cases for space closure, controlling tooth rotations, and extrusion has been intermittently successful, as further proven in a study by Tamer et al. [9]. Clear aligners are driven by two systems. The first is a displacement-driven system, which favors less complicated movements like tipping and control of minor rotations. In this system, aligners are designed based on the proposed final location of the tooth, allowing the tooth to move or displace until it aligns with the aligner. The second is the force-driven system, which operates on biomechanical principles. The amount and type of force applied depend on the shape of the aligners, with each tooth receiving a specific magnitude and type of force as determined by ClinCheck software. This system's aligner details, such as pressure points, facilitate more complex tooth movements like uprighting and intrusion, and power ridges are used to control root torque. In contrast, the former system is better suited for achieving simpler tooth movements [9]. The latter system's effectiveness in complex cases still requires further investigation.

Clinical applications of clear aligners

Scope and Limitations

The biomechanics of clear aligners, as described above, present an interesting phenomenon that forms the basis of their scope and limitations. Understanding these aspects is fundamental to acknowledging the longevity, long-term predictability, and stability of CAT. Numerous studies have been conducted to establish their precise role in orthodontics. However, only a few have successfully discussed their potential functionality and emerging merits, thereby adding breadth to our understanding.

Indications and Contraindications of CAT

The indications and contraindications of CAT are critical for its successful application in orthodontics. The indications of CAT include managing crowding and spacing issues between 1 mm and 5 mm, addressing deep overbites, particularly in class 2 division 2 cases that require intrusion or proclination of incisors, and treating narrow arches that are 4-6 mm due to non-skeletal reasons and need expansion with moderate tipping of the teeth [10,11]. CAT is also indicated for patients with fully erupted permanent teeth, in non-growing patients (late adolescents or adults), and for addressing relapse cases post-fixed appliance treatment. Additionally, it's suitable for tooth movements following inter-proximal reduction (IPR) and staged distalization, as well as for space closure following the extraction of a lower incisor.

Conversely, there are specific contraindications for using CAT [10,11]. These include cases with crowding and spacing greater than 5 mm, skeletal anterior-posterior (AP) discrepancies greater than 2 mm, centric relation (CR) and centric occlusion (CO) mismatches, severely rotated teeth (more than 20 degrees), anterior and posterior open bite cases, extrusion of teeth, severely tipped teeth (more than 45 degrees), teeth with short clinical crowns, and cases involving multiple missing teeth. Understanding these limitations is crucial for the effective and appropriate application of CAT in orthodontic treatments.

This notion has been consistently reiterated: aligners began as a treatment modality for non-growing (adult) patients, mild to moderate malocclusions, and non-extraction cases. A study conducted by Lin et al. shared doubtful results about the effectiveness of CAT as a therapeutic approach for minor orthodontic cases but established that fixed appliance therapy had a greater chance of success in maintaining occlusal contacts [12]. Additionally, CAT showed limited success in tipping second molars for space closure, further confirming its limitations in extraction cases for space fulfillment.

However, in the extant literature, CAT has been studied in growing individuals and is further elaborated on in this review. It has been implicated in moderate to severe malocclusions by utilizing bonded resin attachments on teeth to enhance the scope of the aligners [2]. As Weir et al. further elaborated, the most complex tooth movements cannot occur solely through CAT; they need to be supplemented with auxiliaries and a few geometric changes in aligners [2]. These include the inclusion of bite ramps, pressure points, power ridges for complex root movements, and the addition of inter-maxillary elastics, considering IPR, using temporary anchorage devices (TADs), power arms, and fixed expanders as with fixed orthodontic appliances, to increase the range of the appliance [2].

This is further confirmed by Xing et al., who noted that clear aligners have recently gained popularity in treating extraction cases and have been implicated in tooth rotations, molar distalization, and arch expansion [13]. Further studies on the complicated intrusion and extrusion of teeth concluded that CAT was more efficient in controlling anterior extrusion than intrusion [12]. A modified aligner with a Z spring constructed was also studied for the correction of single-tooth crossbites and became an esthetic, reliable, and cheaper alternative for this purpose [14].

Some cases have been thoroughly assessed to comprehend the scope and applicability of CAT, while others require further investigation and additional evidence to comment on its successful implementation and the potential for long-term treatment stability.

Open Bite Correction

An open bite is characterized by a lack of overlap between the maxillary and mandibular teeth, which can be treated by either extruding the anterior teeth, intruding the posterior teeth, or in some cases, both. As previously mentioned, open bite cases are considered complicated and generally contraindicated for treatment with clear aligners. However, as Proffit mentions, their treatment is possible with the use of auxiliaries and a combined approach [11]. This is supported by a study conducted by El-Bialy, which concluded the same [15]. The hybrid approach, using high-frequency vibration and clear aligners, made it possible to treat a severe case of an adult patient with class III skeletal malocclusion, open bite, and bimaxillary protrusion. The clear aligners successfully moved teeth into the extraction space of an extra premolar found in the lower left quadrant, leading to the resolution of the problem [15]. Another study by Suh et al. investigated the role of CAT in non-extraction cases, finding that approximately 94% of adult patients had their anterior open bite corrected [16]. It was also found that clear aligners led to more vertical control by bringing about maxillary molar intrusion while keeping the position of the mandibular molars intact [16].

Sabouni et al. studied the implication of clear aligners in the treatment of open bite, incorporating three cases: clear aligners alone, clear aligners with attachments and vertical elastics, and clear aligners with attachments and temporary anchorage devices [17]. The research showed satisfactory success in all three scenarios, with auxiliaries treating more complex cases. It emphasized that these types of malocclusions responded better to treatment with clear aligners than fixed appliance therapy because they apply less extrusive force on posterior teeth, which are meant to be intruded in the treatment of open bite. The treatment of open bite with clear aligners involves the combined intrusion of maxillary and mandibular teeth and the combined extrusion of maxillary and mandibular anterior teeth [17]. *Deep Bite Correction* Deep bite, characterized by excessive overlap of maxillary and mandibular teeth, is one of the most complicated cases to treat with clear aligners. Treatment typically involves the intrusion of incisors, extrusion of molars, or both, depending on the incisal show [18]. This process requires careful planning and has achieved limited success with clear aligners. One study found that the use of bite ramps created space for posterior teeth extrusion and anterior teeth intrusion, along with a controlled proclination [19].

Kravitz et al. also confirmed the difficulty in treating deep bite with clear aligners [20]. However, the therapeutic approach has been altered with the incorporation of supporting auxiliaries. These include bite ramps for anterior teeth intrusion and disoccluding the posterior teeth for their extrusion, elastics for posterior teeth extrusion and proclination, and attachments to make aligners more retentive. Additionally, a virtual case setup, which visualizes the forces exerted for particular tooth movements rather than the final tooth position, has proven to be of great importance in planning such cases [20]. Furthermore, aligners designed with specialized intrusion patterns demonstrate varied impacts on the forces applied to different teeth types, such as incisors, canines, and premolars. This variation in force distribution is critical in deep bite correction, as it influences the effectiveness of the aligners in achieving the desired orthodontic movement for each tooth group [21]. There are avenues of exploration that need to be looked into for a reliable and stable outcome.

CAT in mild to moderate cases

Clear aligners have been recognized as effective in treating mild to moderate malocclusions, particularly in managing buccolingual (tipping) movements of upper and lower incisors. However, despite technological advancements, there remains a degree of uncertainty regarding the predictability of outcomes with clear aligners. This calls for more comprehensive research to fully ascertain their efficacy in various orthodontic scenarios [22]. Such studies would help in understanding the limitations and expanding the potential applications of CAT in diverse cases.

Mild to Moderate Crowding Treated with IPR

IPR is a method of gaining space by stripping enamel from tooth surfaces in a pre-determined manner. It plays a crucial role in CAT, as it is necessary for the proper fitting of aligners and the execution of planned tooth movements. In cases of mild and moderate crowding, it is considered one of the potential ways to create space [23]. Although the success of clear aligners in treating crowding has been widely discussed, a study comparing the virtual arch form and the actual arch form post treatment with clear aligners in terms of crowding resolution found the results to be dissimilar. Furthermore, the study revealed that the predictability of crowding relief was 87% in the upper arch and 81% in the lower arch [24]. It also highlighted that IPR, being subject to the operator’s skills, was shown to be an inefficient method for gaining space to relieve crowding.

Mild to Moderate Crowding Corrected with an Arch Expansion

Arch expansion is an effective method to alleviate crowding when there is a discrepancy between arch length and tooth material. The practice of arch expansion using CAT has been examined in various studies. Zhang et al. investigated the effects of arch expansion with clear aligners, focusing on the unintended consequence of buccal flare in posterior teeth [25]. This study emphasized the necessity of applying an appropriate torque compensation angle, considering the patient's current status and compliance, to counteract buccal tipping of the posterior teeth.

The study by Yao et al. delves into the biomechanics of arch expansion using CAT, particularly focusing on the need for controlled torque movements to achieve desired expansion while minimizing anchorage loss [26]. The study emphasizes that precise torque compensation is essential in aligner design to effectively control tooth movement during arch expansion [26]. This is critical in ensuring the stability of the expanded arch and preventing unwanted tooth movements, thereby enhancing the overall effectiveness of CAT in treating mild to moderate crowding through arch expansion. Additionally, a comparison of arch expansion through clear aligners in different planes revealed that transverse arch expansion predictability was 59-83% in the upper arch and 49-67% in the lower arch, decreasing from molars to canines in both arches. In the sagittal plane, predictability remained less consistent [24].

Another study analyzed 15 patients, aged 8-11 years, from January 2020 to December 2021, to examine maxillary and mandibular arch expansion associated with CAT [27]. A three-dimensional (3D) digital oral scanner was used to create a digital model for monitoring progress before and after treatment. It was found that arch expansion was most effective in the maxillary canine region and least effective in the maxillary first molar region. The study suggested that the efficiency of aligners in arch expansion could be enhanced by implementing attachments and desired torque to control tooth movements [27].

Treatment challenges and solutions

Severe Rotations

Bowman et al., in their study, highlight the challenges in correcting dental rotations with clear aligners, especially for severe cases [28]. They note the increased risk of relapse and the need for intricate control, which aligners might not always provide. This suggests the necessity of careful planning and possibly additional orthodontic measures for successful rotation corrections. Adding to this complexity, D’Antò et al. address the difficulties in derotating conically shaped teeth, such as premolars and canines, emphasizing the role of IPR and attachments in enhancing treatment efficacy [29]. Their research also reveals the specific derotation accuracies for molars in class II malocclusions: 77.5% for the first molar and 62.7% for the second molar. These findings collectively underscore the nuanced application of CAT in managing rotational movements and the need for tailored strategies in handling various tooth shapes and severe malocclusions.

Treatment with CAT in Early Years

Sabouni et al.'s research expands the potential use of clear aligners to early transverse, class II, and class III malocclusions in younger patients, challenging the conventional adult-centric application [30]. This exploration into early orthodontics and late mixed dentition phases suggests the versatility of clear aligners. However, the research also highlights significant challenges, particularly regarding the appliance's fit during phases of active dental exfoliation and eruption [30]. This raises concerns about the feasibility and effectiveness of clear aligners in these dynamic oral environments, pointing to a need for further evidence to validate their use in younger patients with ongoing dental development.

*Treatment with Impacted Teeth* The study by Bocchino et al. offers an insightful perspective on treating an impacted maxillary canine using the 'Canine First' approach, combined with CAT [31]. This technique involved surgical exposure and guided alignment of the canine into the dental arch. Following this, CAT was employed to mesially move the canine, substituting for a missing lateral incisor, and achieving space closure. Enameloplasty further refined the tooth's appearance [31]. The study underscores the effectiveness of aligners in aesthetically aligning the canine and the precision in applying force for desired tooth movements. This case illustrates the adaptability of CAT in managing complex orthodontic scenarios, especially in aesthetic zones, highlighting its potential in diverse treatment strategies.

*Premolar Extraction Case* The most common tooth extracted for orthodontic purposes is a premolar. Conventionally, CAT was not considered a suitable treatment regimen for extraction cases, as they were regarded as more complex. However, with the introduction of modifications and attachments, CAT is now being used in such cases. One study presented a case that involved asymmetric extractions in both arches, including premolars and a compromised molar. Clear aligners successfully treated dental crowding and protrusion in a middle-aged patient. However, it was concluded that extraction cases like this require careful planning to overcome undesired movements, such as posterior teeth crowns tipping distally and canine crowns tipping mesially, and there should be extra control over the anterior teeth's torque [13].

Non-extraction cases have been successfully treated with CAT, and recent advances in technology have extended their use to extraction cases. For instance, a study by Tang et al. confirmed the use of CAT in premolar extraction cases [32]. It further compared the anchorage loss when extracting the first and second premolars. The study suggested that evaluating relative anchorage loss is crucial in planning CAT cases [32]. In a recent systematic review by Jaber et al., an extensive literature search encompassed six trials, including three randomized controlled trials, two retrospective cohort studies, and one clinical controlled trial involving a total of 283 patients [2]. This review aimed to assess the effectiveness of clear aligners compared to fixed orthodontic appliances in complex orthodontic cases, particularly those requiring premolar extractions. The findings indicated no significant differences between clear aligners and fixed appliances in certain aspects, as measured by the American Board of Orthodontists Objective Grading System (ABO-OGS) and the Peer Assessment Rating Index. However, discrepancies were observed in some cases between the predicted and actual tooth movements when clear aligners were used in premolar extraction scenarios. Notably, the duration of treatment was generally shorter with fixed appliances than with clear aligners in these instances. Both treatment modalities were effective for premolar extraction-based cases, but fixed appliances achieved better buccolingual inclination and occlusal contacts in a shorter timeframe. The review emphasizes the importance of considering these characteristics when planning orthodontic treatments with clear aligners, especially in complex cases [2].

In a study comparing CAT and fixed appliances treatment (FAT) for treating severe crowding cases requiring premolar extractions, 40 patients were randomized into two groups. The study used the ABO-OGS for evaluation. Results showed no significant difference in overall effectiveness between CAT and FAT, although fixed appliances were slightly better in occlusal contacts. Success rates were similar in both groups, with fixed appliances having a marginally higher success rate. This suggests both treatments are viable for complex orthodontic cases, with some advantages in fixed appliances [33].

Molar Distalization

Molar distalization has always posed challenges in CAT, yet recent advancements have not ruled out its feasibility. CAT has evolved to become a pivotal component in treating class II malocclusions, necessitating maxillary molar distalization. This technique is increasingly seen as a valid non-extraction alternative, offering reduced maxillary molar extrusion and enhanced occlusal and vertical control [34]. D’Antò et al. affirmed CAT's efficacy in molar distalization, observing successful movements of 2-3 mm without losing control [29]. Their study reported an overall accuracy of 69.3% for the first molar and 75.2% for the second molar, utilizing refinements and attachments. Furthermore, recent studies demonstrate CAT's capacity to distalize mandibular molars effectively, particularly for corrections over 2 mm in the sagittal plane, using mini-implants [35]. This expands CAT's utility to more complex mandibular movements, thus broadening its orthodontic applications. The discrepancy observed between anticipated and actual outcomes, especially when anterior retraction is involved, highlights the intricacies of biological responses in orthodontic treatments [36]. Lower efficacy rates in cases involving simultaneous anterior retraction suggest potential hindrances in molar distalization, emphasizing the necessity for meticulous planning in concurrent orthodontic movements. The notable arch expansion observed may serve as a compensatory anatomical response, bearing functional and aesthetic consequences. These findings underscore the significance of tailored treatment strategies and the potential need for adaptable approaches throughout CAT.

Patient-centric factors in CAT success

Long-Term Treatment Stability

Many patients favor CAT as they perceive it to be the most socially acceptable management strategy for their orthodontic issues. However, they often have limited knowledge about the clinical factors underpinning this decision. Only a few studies have comprehensively addressed the detailed efficacy of clear aligners, with the majority still striving to establish a firm conclusion. This review aims to delineate some of the key determinants in the long-term stability of CAT and to explain how each factor contributes to the reliability of the appliance over time.

Orthodontic Case Selection

To achieve the treatment goals with CAT and to ensure the long-term stability of the adjustments undertaken, it is essential to select the right case for it. Clear aligners have been used for the treatment of less complicated malocclusions. Undoubtedly, in most complex cases conventional orthodontic treatment with braces is the unmatched option to date. As confirmed by the investigation comparing different perspectives about CAT in orthodontists and general dentists, 45% of the orthodontists were reluctant to treat patients using clear aligners because of their limited efficacy in certain cases, and 40% of general dentists were discouraged from doing so due to their inexperience [37].

One systemic review conducted by Rosini et al. analyzed certain research done in the past to state the efficacy and predictability of CAT to allow different orthodontic movements in non-growing patients [38]. It was found that it is an effective clinical regimen when used for alignment and leveling of the arches, the intrusion of anterior teeth was found to be the same as with conventional fixed appliances. The complexity of tooth movements increased for CAT against anterior teeth extrusion and rotation and it did not prove to be beneficial for such tooth movements.

Following the same approach, different studies were undertaken for this review to identify the case-to-case variation to learn about the true potential of CAT. Borda et al. studied the efficacy of CAT in comparison to fixed orthodontic braces for the treatment of mild malocclusion in teenage patients [8]. Affirming the research, it was concluded that interproximal and occlusal contacts, the position of the marginal ridge, and the buccolingual inclination of the teeth, with CAT were analogous to fixed orthodontic therapy. The overall results for mild malocclusion were a great success for CAT. Chou et al. conducted a study on adolescents with moderate to severe class I and II malocclusions, comparing CAT with FAT [39]. The results demonstrated that both treatment modalities were effective, but CAT showed a marked efficiency advantage. On average, CAT treatments were completed three months faster and required eight fewer visits than FAT, highlighting CAT's time efficiency in managing these malocclusions [39]. These findings illustrate the potential of CAT as a more time-efficient option in treating complex cases in adolescents, making it a considerable choice in orthodontic case selection.

Little evidence exists about moderate to severe cases being treated by CAT which has been discussed in this review and needs further evaluation, so orthodontists select the right fit for the treatment provision to safeguard the long-term stability of the cases that will most definitely benefit from it.

Patient Compliance

Patient adherence is pivotal for the success of CAT, as emphasized in recent studies [5,10]. These studies specify the necessity of wearing aligners for a minimum of 20-22 hours daily, translating to about 400 hours per aligner for effective treatment. The onus of compliance lies heavily on the patient due to the removable nature of aligners, increasing the risk of distortion or loss, especially given their clear appearance. Adherence to aligner wear and maintenance is crucial in younger patients, who might not be ideal candidates. Non-compliance, including inadequate wear time, missed appointments, and poor oral hygiene, can severely undermine treatment outcomes, regardless of case simplicity [5,10]. Hence, consistent adherence to CAT protocols is a decisive factor in ensuring the long-term efficacy and stability of the treatment, as highlighted by the data and insights from these studies.

In a randomized clinical trial, Jaber et al. compared the impact of CAT and FAT on oral health-related quality of life in patients with severe crowding [40]. The study revealed that clear aligners had a less negative impact on patients' quality of life compared to fixed appliances. This was particularly evident in aspects such as functional limitation, physical pain, and physical disability. Notably, clear aligners resulted in fewer issues related to eating, pronunciation, and general discomfort, especially during the early stages of orthodontic treatment.

Patient’s Skeletal Growth

Recent research by Staderini and colleagues explores the utilization of CAT in growing patients, traditionally a domain for non-growing individuals [41]. The study, focusing on early treatment of anterior crossbite using CAT, shows promising yet preliminary results in young patients. Growing individuals, it appears, are more responsive to CAT, offering comfort and quicker solutions compared to braces. However, challenges such as ongoing growth affecting tooth movement and relapse risk remain. The study highlights CAT's acceptance in mixed dentition, prioritizing patient comfort and rapid treatment over detailed outcome knowledge. Understanding a patient’s growth and skeletal maturity is vital in predicting treatment results, emphasizing the importance of careful case selection in growing individuals using CAT [41]. A comprehensive understanding of a patient’s growth status and skeletal maturity indicators can be crucial in predicting treatment outcomes in advance. Proper case selection is key to ensuring the long-term predictability of CAT in growing individuals.

Identification of Individual Variability

For an orthodontist, adopting the mindset that 'every case is a new case' is crucial when managing different patients. Given the biological and genetic uniqueness of each individual, a predictable outcome is not always guaranteed. However, it can be made more likely by considering the necessary prerequisites, thereby enhancing the chances of long-term success. Numerous studies have explored various variables; for example, the role of attachments has been widely studied, with findings indicating their positive impact on facilitating complex tooth movements. One study demonstrated a 70% efficacy in achieving maxillary transverse expansion without using any auxiliaries [42]. While many cases can be effectively treated with clear aligners alone, others, such as premolar extraction cases, may require combination therapy to achieve complex tooth movements like bodily movement, extrusion, root torquing, etc. [43]. This illustrates how case-to-case variation and a patient’s adaptability can contribute to a successful outcome. Furthermore, a comprehensive analysis has revealed that the polyurethane plastic material used in clear aligners significantly influences their mechanical properties and force application. Over time, these aligners are susceptible to mechanical degradation due to intraoral aging, leading to a decrease in force exertion and, consequently, a decline in their therapeutic efficacy. Additionally, the use of attachments with clear aligners can intensify this deterioration, potentially affecting the surface integrity of the appliance. This aspect is critical in understanding individual variability in treatment outcomes and underscores the importance of material considerations in aligner effectiveness [44].

Yaosen C et al.'s research underscores the impact of individual variability in orthodontic treatment with clear aligners [45]. Factors such as unilateral chewing habits can lead to the detachment of aligner attachments, highlighting the influence of personal behaviors. Additionally, the effectiveness of clear aligners is affected by individual oral hygiene, saliva composition, and dietary habits. Therefore, it's vital for orthodontists to engage in detailed, personalized assessments, tailoring treatment strategies to each patient's unique needs. This approach is crucial for achieving lasting, effective outcomes in orthodontic care.

Long-term stability and retention strategies

Anchorage Planning

Are clear aligners reliable substitutes for fixed orthodontic therapy? Given the inadvertent need for torque to elicit complex tooth movements and anchorage planning [46], the answer is neither straightforward theoretically nor practically. Anchorage planning is crucial for stability and resistance to unwanted movements of the anchor unit. In contrast, anchorage planning in fixed appliances is quite successful due to their metallic construction and firm adhesion. However, clear aligners, being removable entities made of thin plastic material, invariably require auxiliaries for anchorage planning and to prevent undue tooth movements. A study focusing on the use of attachments and advancements in aligner materials indicated that these measures have not completely resolved the issue, and anchorage loss remains a problem in the long run with CAT [46]. Liu et al. conducted an in-depth analysis of the use of class II elastics in maxillary molar distalization using CAT [47]. Their findings underscored the effectiveness of class II elastics in enhancing anchorage, significantly limiting maxillary incisor proclination and canine extrusion. The study demonstrated that, with the use of class II elastics, there was a notable reduction in the forward movement of the maxillary anterior teeth, indicating a more controlled and effective approach to anchorage management. This research provides valuable insights for anchorage planning in CAT, highlighting the role of supplemental techniques in achieving desired orthodontic outcomes [47]. Therefore, careful anchorage planning with CAT can signify a change in the course of treatment and ensure long-term stability.

Retention Phase

The long-term stability of orthodontic treatment is determined by the type of tooth movement, the shift induced, the duration of active treatment, and the post-treatment retention regimen. The retention phase is crucial for preserving the changes made during active treatment, as teeth have a tendency to revert to their original position. Relapse can occur due to the violation of the neutral zone. It is important to note that mandibular arches are less stable than maxillary arches, so their expansion should be carefully planned. Additionally, continual growth changes in the inter-canine width due to aging can contribute to instability [48].

The importance of adherence to the retention phase by patients is critical for long-term efficacy and overall clinical success. Various studies have aimed to differentiate the relapse occurring from FAT and CAT. It has been theorized that relapse is more common in treatments with clear aligners than with fixed appliance therapy following active treatment and a specific retention period. Graf et al. investigated the impact of retention on treatment outcomes with a clear aligner over a period of 10 months [49]. In their study, the mandibular arch was fitted with a bonded lingual retainer from canine to canine, and a removable Hawley’s retainer was used in the maxillary arch. The study concluded that the retention and stability of achieved tooth movements were maintained 10 months post retention. Graf also pointed out the risks of overcorrection and exceeding physiological boundaries, which can alter natural arch forms and make changes less stable in the long run [49], highlighting the need for strict adherence to retention protocols, which may not always be favored by patients. The frequent incidence of relapse after CAT underlines the importance for patients to follow the protocol and for orthodontists to meticulously plan both complicated and less complicated cases, as well as the post-treatment retention phase. This ensures the time, effort, and money spent on CAT are worthwhile. Alongside routine retention appliances and devices used in clinics, minor operational procedures such as IPR and high cusps for correct interdigitation in buccal segments, which are beneficial in all three dimensions (sagittal, transverse, and vertical), can also be incorporated for final occlusal adjustments, leading to more stable outcomes [48].

Orthodontic expertise and treatment planning

Orthodontist’s Role and Decision-Making

The ability of orthodontists to establish a definitive diagnosis and proceed with an accurate treatment plan for CAT is crucial, as it directly impacts the success and long-term stability of the case. The importance of precise case planning for achieving desired outcomes and its relation to the long-term efficacy of CAT is exemplified in a study by Smith et al. [50]. This study focused on a case of lower incisor tipping using CAT. In the crowded anterior mandibular region, there is often a need for root uprighting. Initially, the root movement was less than predicted, but with the orthodontist's timely decision to incorporate vertical rectangular attachments into the appliance for greater root movements, the outcome became predictable [50]. This review has highlighted the inclusion of attachments and auxiliaries in CAT to extend its capabilities. Expertise in planning and applying these attachments is essential.

In their comprehensive study, Perillo et al. found that orthodontists generally have more experience with clear aligners than general dentists [37]. This difference was significant, with a greater percentage of orthodontists using clear aligners in their practice. Furthermore, orthodontists treated more class I malocclusions with crowding and open bite than general dentists [37]. Another study, focusing on treatment management in CAT, substantiated that orthodontists are more inclined to employ auxiliary tools and supplemental techniques compared to general dentists. The study revealed significant differences in treatment management strategies, with orthodontists being notably more likely to utilize various auxiliaries such as elastics (92% vs. 37% in general dentists for class II elastics), extractions, and a combination of fixed appliances with Invisalign. These findings highlight the complex and nuanced decision-making process inherent in orthodontists' approach to CAT, underscoring their specialized expertise in managing diverse orthodontic cases [51].

Sabouni et al. showcased a complex case involving a 25-year-old female patient with a class I skeletal relationship, bilateral class II dental relationship, increased overjet, deep bite, and crowded maxillary and mandibular arches [52]. This case was considered among the most challenging to treat according to available literature. However, the study concluded that appropriate attachment selection was an effective means of treating such cases [52]. The expertise demonstrated in this study, particularly in the judicious selection and application of attachments, is highly appreciated and serves as a testament to the skillful and innovative approaches required in advanced orthodontic treatments like CAT. Moreover, the expertise of orthodontists in carefully selecting cases, coupled with a step-by-step approach to molar distalization, precise anchorage planning, and the use of class II elastics, confirms the long-term stability of CAT in complex cases. As advancements continue, the scope of CAT broadens.

Treatment Monitoring and Follow-up

The orthodontist’s expertise is crucial in pre-treatment, mid-treatment, and post-treatment decision-making. Situations often deviate from the planned course of treatment, requiring the orthodontist to make timely and accurate decisions to ensure successful outcomes in cases selected for CAT. The randomized clinical trial by Al-Nadawi et al. exemplifies the importance of follow-ups and adjustments to the original plan [53]. They studied the effects of different CAT wear protocols on treatment outcomes at seven-day, 10-day, and 14-day intervals. The study found that all linear discrepancies were insignificant (<0.5 mm), but angular discrepancies remained significant (> 2.0 degrees). Comparable accuracy was observed between the 7-day and 14-day protocols, favoring shorter treatment durations. However, it was noted that posterior movements such as torque, tipping, and rotation required a longer 14-day duration. Mid-treatment follow-ups and routine examinations to predict different regimens positively impact the long-term stability of CAT [53]. Regular follow-up visits are essential to predict favorable outcomes, allowing monitoring of developments in the case and early intervention for any complications. This approach saves time and enhances efficiency in the long run.

When planning a case, orthodontists must anticipate changes at the core of the problem. Recent innovations have facilitated this, proving beneficial for planning and monitoring cases. A study comparing software-predicted outcomes with actual clinical outcomes from aligner stages T0 to T4, T0 to T6, and T0 to T8 showed significant variations, with T4 accounting for 62% accuracy, T6 for 68%, and T8 for 78% in correction. This concluded that CAT was effective against mild to moderate malocclusion, but the success rate could be improved beyond what was predicted by proprietary software models [54]. Another study investigated serial digital scans for orthodontic tooth movements to record results consistently. Cases involving root movements in CAT are gaining attention for two reasons: first, the occlusion is ultimately influenced by root tipping; second, this affects the final fit of the aligner. If not as planned, the aligner’s mechanical properties risk influencing the biomechanics of the appliance, leading to uncertain long-term stability [50]. Mao et al. conducted a study on simulated tooth movements in molar distalization cases, examining the effect of maxillary molar distalization with clear aligners using finite element methods (FEM) models of maxillary dentition, attachments, periodontal ligaments, and the specified aligner morphology [55]. The study during the staged distalization process noted anterior teeth proclination and distal tilting of the second molar.

The growing popularity of innovations and software development has simplified predicting clinical outcomes, enabling the identification of adverse occurrences and risk factors earlier to ensure a sustained treatment regimen.

Material science in clear aligners

Aligner Material

An ideal aligner material for CAT should have low hardness, high resilience, adequate elasticity, resistance to warpage, good biocompatibility, and optimal transparency. Advances in CAT materials have introduced a variety of options, including single entities, blends of different materials, shape memory polymers, 3D printed materials, and bioactive materials, enhancing the effectiveness and long-term stability of clear aligners [56]. The material used in aligners should be appropriately stiff. Overly stiff materials can complicate placement and removal, while too little stiffness might not provide adequate force for tooth movement. The viscoelastic nature of these materials helps in absorbing forces, enabling effective force delivery to the teeth. However, material transparency can diminish due to wear from eating and drinking, impacting aesthetic appeal [56].

Multi-layer clear aligners in orthodontics provide several benefits compared to single-layer versions. They are more effective in tooth movement, better at distributing stress on the periodontal ligament, and more efficient in load distribution on the alveolar bone, particularly those with a higher soft-to-hard layer ratio [57]. These aligners consist of a hard outer thermoplastic layer, a softer middle layer, and a reinforced resin core, offering a balance between wear resistance, mechanical strength, and occlusal force distribution [57]. The soft layer increases elasticity and impact absorption, while the hard outer layer maintains shape and structural integrity. Furthermore, multi-layer aligners have lower water absorption rates than double-layer types, suggesting enhanced durability and intraoral stability [57].

Overall, while maintaining ideal material properties is crucial for the normal functioning of clear aligners, the introduction of multi-layer designs represents a significant advancement in aligner technology, promising more predictable outcomes in orthodontic treatments.

## Conclusions

CAT has established itself as a prevalent choice in contemporary orthodontics, particularly for mild to moderate malocclusions. This narrative review underlines that while CAT shows promising results in these cases, it is also progressively addressing more complex scenarios and challenging tooth movements. Key determinants of long-term treatment stability, such as patient compliance and professional orthodontic expertise, are critical. An orthodontist's adeptness in case selection, meticulous planning, utilization of advanced technologies and software, individualized patient monitoring, and effective execution of retention protocols are instrumental in achieving sustained success with CAT. Furthermore, a thorough understanding of aligner material properties significantly contributes to treatment efficacy. There is a pressing need for ongoing research to expand our understanding of CAT's capabilities and limitations, which will enhance its application scope and optimize outcomes in diverse orthodontic scenarios.
